# Long Distance Consultations

**Published:** 1913-06

**Authors:** 


### CORRESPONDENCE.

*This department is intended for the presentation of news and views not coming within the scope of other departments, and particularly to afford opportunity for discussion and criticism of views elsewhere expressed.*

---

### Long Distance Consultations.

April 16, 1913.

Buffalo Medical and Surgical Specialists, Buffalo, N. Y.:

Dear Sirs—Having been in poor health and as my son doctored with you a number of years ago, I thought I would write you. I have been poorly about six or eight months. If you think you can do me any good I would like to hear from you right away, for I need some aid. I am able to be up but can't work any. Write me what you think about me. Yours respectfully,

——— ———.

---

Dear Sir—Your letter was delivered to this journal. There are so many medical and surgical specialists in the city that it would be useless to try to forward the letter. If the postoffice supplied an address it would undoubtedly go to some quack firm advertising under a similar title. Our advice to you is not to write to any such firm and not to try long-distance treatment even by a physician in good standing, but to consult personally some local physician or, if you feel that you need some special degree of skill, to ask him to refer you to the appropriate specialist in some city near you. Very truly yours,

——— ———.

---

My Dear Doctor:

If you meet with any cases of cancer, I wish you would please refer them to me. I have made a business of the medical treatment of cancer for — years. My book —— is now used on both sides of the Atlantic ——— gives a record of 100 cases of all forms and how I cured them, also it gives 75 remedies that are curative in cancer ———.

The above is not a circular letter, but was addressed to the editor personally. As the writer is not a personal acquaintance, we may be exculpated of the charge of being a cad for printing these extracts, if not of selfishness and prejudice in holding on to such cases as our surgical friends consider inoperable, and of ignorance in confessing that we do not know of a single remedy that is anything like curative in cancer. But we would give $1000 to have such a case of cholesclerosis.

